# Projected Augmented Reality in Surgery: History, Validation, and Future Applications

**DOI:** 10.3390/jcm14228246

**Published:** 2025-11-20

**Authors:** Nikhil Dipak Shah, Lohrasb Sayadi, Peyman Kassani, Raj Vyas

**Affiliations:** Department of Plastic and Reconstructive Surgery, University of California, Irvine, CA 92697, USA

**Keywords:** augmented reality in surgery, intraoperative visualization, projection mapping in medicine, 3D surface registration, surgical navigation technology, craniofacial surgery innovation, medical education technology, global surgery and capacity building, mixed reality in healthcare

## Abstract

**Background/Objectives:** Projected augmented reality (PAR) enables real-time projection of digital surgical information directly onto the operative field. This offers a hands-free, headset-free platform that is universally visible to all members of the surgical team. Compared to head-mounted display systems, which are limited by restricted fields of view, ergonomic challenges, and user exclusivity, PAR provides a more intuitive and collaborative surgical interface. When paired with artificial intelligence (AI), PAR has the potential to automate aspects of surgical planning and deliver high-precision guidance in both high-resource and global health settings. Our team is working on the development and validation of a PAR platform to dynamically project surgical and anatomic markings directly onto the patients intraoperatively. **Methods:** We developed a PAR system using a structured light scanner and depth camera to generate digital 3D surface reconstructions of a patient’s anatomy. Surgical markings were then made digitally, and a projector was used to precisely project these points directly onto the patient’s skin. We also developed a trained machine learning model that detects cleft lip landmarks and automatically designs surgical markings, with the plan to integrate this into our PAR system. **Results:** The PAR system accurately projected surgeon and AI-generated surgical markings onto anatomical models with sub-millimeter precision. Projections remained aligned during movement and were clearly visible to the entire surgical team without requiring wearable hardware. **Conclusions**: PAR integrated with AI provides accurate, real-time, and shared intraoperative guidance. This platform improves surgical precision and has broad potential for remote mentorship and global surgical training.

## 1. Introduction

Over the past decade, the accessibility and affordability of mixed reality systems have accelerated their adoption across diverse domains, ranging from entertainment and architecture to defense and healthcare. Mixed reality encompasses technologies that merge digital and physical environments, most prominently virtual reality (VR) and augmented reality (AR). VR fully immerses the user in a computer-generated environment, replacing all real-world sensory input with simulated audiovisual stimuli. AR, on the other hand, overlays digital content onto the real world in real time, registering virtual elements to the user’s actual environment without occluding it [1]. Within AR, head-mounted display augmented reality (HMD-AR) and projected augmented reality (PAR) represent the two dominant approaches, each with distinct user experiences and clinical implications.

HMD-AR, exemplified by products such as Microsoft HoloLens and Apple Vision Pro, has gained cultural visibility through consumer markets. In medicine, HMD-AR has been investigated for tasks such as anatomic visualization, preoperative planning, and simulation [2,3,4]. For example, HMD-AR has been used to visualize patient-specific radiology (CT/MRI) to overlay cranial tumors or vascular malformations for neurosurgery and acetabular and distal radius fracture maps for orthopedics. Additionally, HMD-AR has supported task rehearsal and skills acquisition in endoscopic skull-base and sinus surgery and laparoscopic surgery by projecting stepwise holographic guidance and objective performance metrics onto physical trainers or mannequins. Overall, HMD-AR has advanced markedly, with platforms such as HoloLens 2 and Vision Pro offering wider fields of view, improved optical see-through, and hand/eye tracking, all of which support credible clinical integration. However, widespread intraoperative adoption remains limited. Barriers include the inability to wear headsets simultaneously with loupe magnification, limited fields of view, and exclusivity of visualization to the headset wearer. These ergonomic and perceptual challenges help explain the limited integration of HMD-AR into the operating room.

By contrast, projected augmented reality (PAR) projects images directly onto the operative field, offering a complement to the already established HMD-AR [5]. PAR offers a larger field of view and universal visibility for all team members. Most importantly, it places information precisely where it is needed: directly on the patient’s anatomy. This promotes seamless integration, allowing the technology to fade into the background while augmenting decision-making.

The clinical promise of PAR lies not only in enhancing surgical precision in high-resource environments, but also in democratizing surgical expertise globally. At a time when more than five billion people lack access to safe, affordable surgery, PAR offers a mechanism to scale expertise, enabling real-time remote guidance and sustainable capacity building in underserved regions. This review explores the historical development of PAR, its technical underpinnings, clinical validation, and emerging applications, with particular focus on its transformative role in surgical education and global health.

## 2. Development of Augmented and Projected Augmented Reality

### 2.1. Early Concepts of Augmented Reality

**Figure 1:**
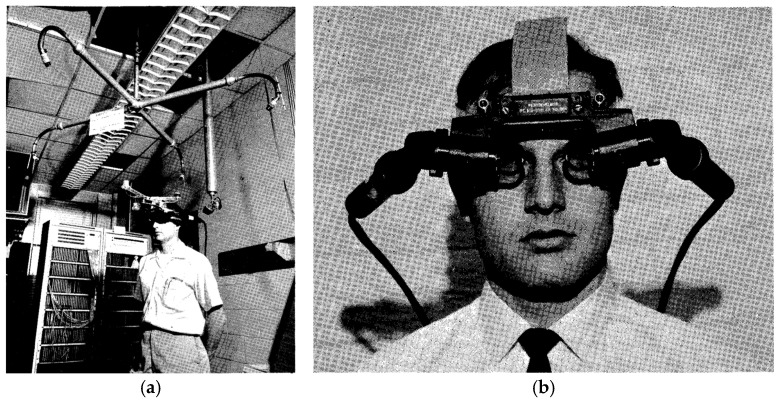
(**a**) Head-mounted display system created by Dr Ivan Sutherland (**b**) Head-mounted display optics (Adopted with permission from the Association for Computing Machinery).

The origins of AR trace back to 1968, when Ivan Sutherland and colleagues developed the first head-mounted display system, colloquially known as the “Sword of Damocles.” This rudimentary platform was suspended from the ceiling and displayed basic wireframe graphics that responded to the user’s head movements (Figure 1). While primitive, it introduced the principle of overlaying computer-generated content onto real-world views.

As computing power expanded through the 1980s and 1990s, AR applications proliferated, though primarily in defense, aviation, and industrial domains. The evolution of compact sensors, miniaturized processors, and advanced displays enabled consumer-grade AR devices in the 2010s, spearheaded by Google—Google Glass (Mountain View, CA, USA), Microsoft—HoloLens (Redmond, WA, USA), and more recently Apple—Vision Pro (Cupertino, CA, USA). These advances solidified HMD-AR as the dominant paradigm for personal augmented experiences.

### 2.2. Emergence of Projected Augmented Reality

Parallel to the trajectory of head-mounted displays, projected augmented reality (PAR) emerged as a distinct branch of AR with a fundamentally different philosophy. Rather than confining digital content to a headset worn by the user, PAR leverages projection mapping to cast imagery directly onto physical surfaces in the environment. Projection mapping is a computer-vision–guided technique that warps and aligns projected imagery to the exact geometry of a physical surface, so the light conforms to contoured regions without distortion. In effect, the surgical field, building façade, or battlefield map itself becomes the display, eliminating the need for personal headgear and creating a universally visible, shared visualization.

The conceptual roots of PAR can be traced to Disneyland in 1969, when filmed performances were projected onto animatronic figures to create the illusion of lifelike movement and expression. The scientific foundations were formalized in the early 2000s by Raskar and colleagues, who introduced the concept of “spatially aware projectors.” These were systems capable of sensing their position relative to a surface and dynamically warping their output to conform to complex geometries [5,6]. This breakthrough, along with advances in structured light scanning, dynamic projection mapping, and multi-projector calibration, made it possible to project accurately onto irregular, curved, and even deformable surfaces.

**Figure 2:**
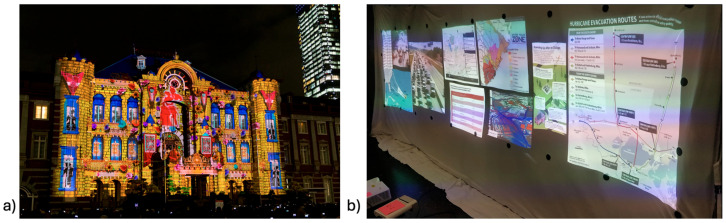
(**a**) “Projection Mapping to Tokyo Station” by t-mizo is licensed under CC BY 4.0 (**b**) Projection mapping on tent surface in a military setting (https://www.summittechlab.com/federal, accessed on 4 April 2025).

Once technically feasible, PAR was rapidly adopted across multiple industries. In the military context, the U.S. Air Force deployed large-scale projection systems to display interactive battlefield maps across tent walls, allowing commanders and troops to collaboratively plan missions with shared situational awareness. Commercially, projection mapping became a cornerstone of immersive entertainment, from live concerts and theater productions to advertising campaigns that transform building exteriors into dynamic storytelling canvases (Figure 2). Architects and designers integrated PAR into client presentations, projecting interactive renderings of proposed structures at full scale to convey spatial relationships more vividly than drawings or digital models alone. In industrial logistics, projection-guided workflows have been trialed to optimize warehouse operations, guiding workers with dynamic overlays projected directly onto shelves or workstations. Robotics research has also employed PAR to improve human–robot collaboration, projecting intent, pathways, or hazards directly into shared physical space [7,8,9,10].

Together, these precedents underscore the scalability, robustness, and collaborative potential of PAR as a visualization paradigm. Its proven performance in high-stakes military planning, high-throughput industrial environments, and large-scale commercial venues provides strong evidence of its adaptability. These qualities make PAR particularly well suited to surgery, where visualizations must be intuitive, real-time, and sharable among an entire operative team. The translation of these principles into the operating room thus represents not a speculative leap, but a natural extension of a technology already tested in some of the most demanding environments outside of healthcare.

### 2.3. Transition into Medicine

While AR’s medical applications initially centered on HMD-based visualization of preoperative imaging, PAR entered healthcare later, coinciding with improvements in surface scanning, dynamic calibration, and compact projection systems. PAR is well-suited for surgery, where information must be integrated onto the operative field, shared among all team members, and must be continuously adaptable to tissue deformation. These attributes position PAR as a compatible platform for intraoperative guidance, offering intuitive, field-based visualization without the ergonomic burdens of head-mounted displays.

Within clinical practice, PAR’s best-known use is near-infrared vein mapping, where hemoglobin absorption patterns are projected onto skin to facilitate venipuncture [11]. More advanced implementations include hepatic surgery, where indocyanine green fluorescence can be projected onto the liver to delineate blood flow and which segments a surgeon should resect [12].

PAR has also proven valuable in medical education. Systems have projected anatomic structures onto mannequins, cadavers, and even live students, dramatically enhancing spatial understanding of complex anatomy [13,14]. Unlike textbooks or screen-based 2D simulations, PAR provides direct, full-scale visualization on tangible surfaces. Learners can see and interact with anatomy in its true dimensions, bridging the gap between abstract conceptual knowledge and embodied practice. By externalizing digital information into the learner’s physical environment, PAR reinforces spatial learning in ways that traditional teaching modalities cannot.

Together, these applications underscore the natural fit of PAR for medicine. In the operating room, it provides real-time, universally visible guidance anchored directly to the patient. In the classroom or simulation lab, it transforms static learning into immersive experiences that prepare trainees for the complexity of clinical practice.

## 3. Validation of Augmented Reality in Surgical Guidance

### 3.1. Remote Transfer of Surgical Expertise

AR is recognized as a tool for remote surgical teaching. Vyas and colleagues demonstrated that tablet-based AR could be used to proctor cleft lip repairs in Peru, enabling U.S. experts to provide remote, real-time intraoperative guidance. In this study, continuous live audio/video of a cleft lip procedure was transmitted overseas while a remote surgeon was able to interact with the surgical field via an augmented reality platform. Appropriate markings were then displayed on a screen in the operating room in Peru. After 30 months, local surgeons achieved independence in cleft care, eliminating the need for patient transfers to distant tertiary centers [15]. However, this system required learners to translate markings from a screen to the patient, introducing risk of error. PAR circumvents this limitation by projecting expert input directly onto the operative field and allowing for intraoperative guidance. This transition, from indirect visualization to direct projection, represents a critical step towards increasing precision in surgical mentorship.

### 3.2. Proof-of-Concept Studies in Surgery

Several groups have explored projection-based augmented reality (PAR) to address specific intraoperative challenges, each focusing on a different element of reconstructive surgery [16,17,18,19]. Cifuentes et al. introduced PAR for vascular mapping by using portable projectors to overlay thermographic data on flap donor sites. By projecting heat maps directly onto the anterolateral thigh, they were able to identify perforator blood vessels crucial for flap viability, effectively translating non-visible physiologic data into real-time, intuitive guidance for flap harvest [16]. Sayadi et al. advanced this concept in flap design by projecting local flap templates onto cutaneous defects. In a comparative analysis, they measured the geometric accuracy of projected templates against surgeon-drawn freehand markings, finding that projection yielded superior angular precision, side length proportionality, and overall reproducibility, an important step toward standardizing soft tissue reconstruction [18,20]. Schreiber et al. applied PAR to fat grafting, focusing on the periorbital region where millimetric accuracy is essential. This system allowed projected overlays to guide injection trajectories and distribution patterns, providing a visual scaffold that improved precision and symmetry during graft placement [17]. Lastly, Hummelink et al. demonstrated how preoperative computed tomographic angiography (CTA) could be integrated into PAR workflows. By projecting the course of deep inferior epigastric skin perforators directly onto the abdominal skin of patients undergoing breast reconstruction, their method provided a skin-level map of subsurface vascular anatomy, reducing exploratory dissection and potentially shortening operative time [19]. Collectively, these studies illustrate the adaptability of PAR across a spectrum of reconstructive tasks ranging from flap harvest to cutaneous design and grafting. Of note, these studies are all single-center, have small sample sizes and nonrandomized designs, and rely on technical surrogates rather than patient outcomes. This highlights the need for larger, reproducible trials with standardized clinical endpoints prior to clinical application.

Although these proof-of-concept studies validated the feasibility of PAR, they also revealed technical and translational limitations that must be addressed before routine clinical adoption. A central challenge is the lack of compensation for contour distortion, as all four systems projected two-dimensional data onto complex three-dimensional anatomy without surface-aware correction. This constraint reduces overlay accuracy when applied to curved or irregular topographies, such as the periorbital region or nasolabial complex, where surgical margins depend on sub-millimeter precision. Furthermore, the studies largely operated under controlled conditions, with limited discussion of how projection would withstand intraoperative variables such as movement and ambient light. No models incorporated real-time updates to accommodate tissue deformation during dissection. Similarly, Sayadi et al.’s work demonstrated the value of projection for teaching geometric consistency but did not tackle challenges of dynamic motion or surgeon-driven customization in the operative field. Thus, while these early demonstrations laid important groundwork by proving that PAR can be applied safely and usefully to surgery, they underscored the need for next-generation systems that integrate three-dimensional surface scanning, dynamic projection mapping, and adjustable landmarking to achieve clinically robust precision.

### 3.3. Integration with Structured Light Scanning

**Figure 3:**
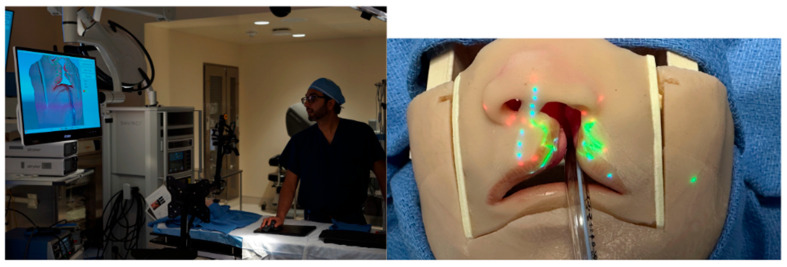
Real-time cleft lip markings displayed via PAR on Simulare Cleft model by Dr Raj Vyas.

To overcome the challenge of three-dimensional tissue distortion, PAR can be coupled with structured light scanning (SLS), a technology that enables precise digital capture of complex surface anatomy. In SLS, a projector sequentially casts coded light patterns across the surgical field and a co-registered camera records the way these patterns deform as they strike curved or irregular surfaces. Computational decoding of these distortions yields a high-density three-dimensional mesh of the operative site with sub-millimeter precision between the digital and physical surfaces. This 3D model serves as the geometric “canvas”, allowing projected markings to be warped in real time so that they conform to local topography rather than skewing across a complex three-dimensional surface (Figure 3).

**Figure 4:**
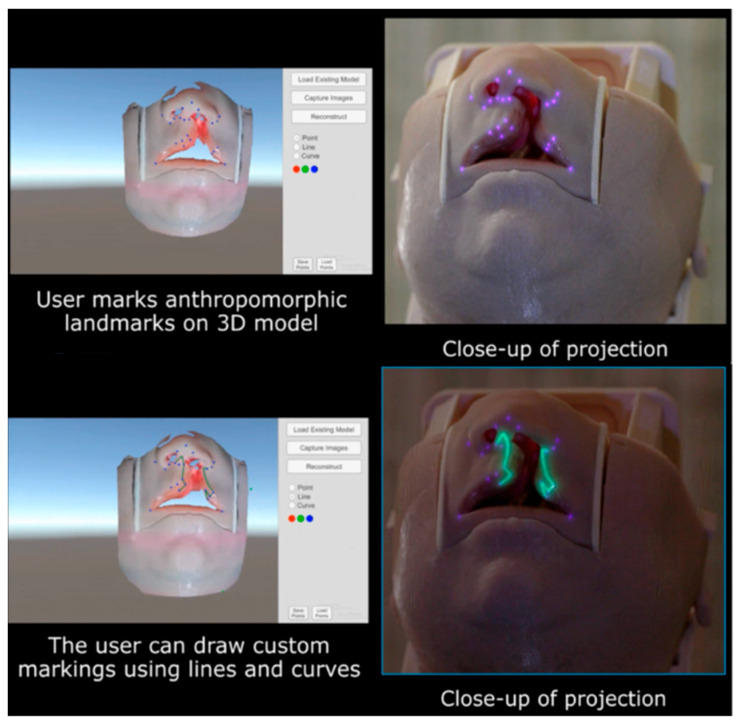
Annotations made on 3D digital model of a surgical site by a remote surgeon (**left**) would be projected directly onto the patient’s surgical site in real-time using PAR (**right**). **Above**: 21 cleft anthropometric points. **Below**: Mohler cleft lip repair markings.

Our group has directly applied this principle to cleft lip models, where the small footprint and intricate curvature of the nasolabial region provide a particularly stringent test of accuracy. Using SLS, we reconstructed a faithful 3D representation of the infant lip and nose and then superimposed anthropometric landmarks and full surgical designs, such as the Mulliken repair pattern, directly onto the model surface (Figure 4). The system ensured that projected markings hugged the convexity of the philtrum, columella, and alar base, preserving fidelity across slopes and inflections that would otherwise distort flat projections. Importantly, real-time depth cameras allowed continuous updating of the mesh, so that when the head was rotated or tilted, simulating intraoperative repositioning, the projected design automatically shifted and re-registered without lag (Supplemetary Materials Video S1).

Beyond local accuracy, this workflow introduces a powerful mechanism for remote surgical collaboration. Once an SLS scan is acquired, the 3D mesh can be transmitted to experts anywhere in the world. Remote educators or consultants can digitally annotate landmarks, incision lines, or flap templates on the mesh, and then return the annotated file to the operative team. Locally, the PAR system translates these remote markings back onto the live surface, where they are projected directly on the patient. This creates a closed-loop system of capture, remote annotation, and re-projection, enabling geographically distant experts to “mark” a surgical field as if they were standing in the operating room. In this way, the integration of SLS with PAR transforms static projection into a dynamic, distributable platform for real-time surgical guidance.

### 3.4. Artificial Intelligence Integration

**Figure 5:**
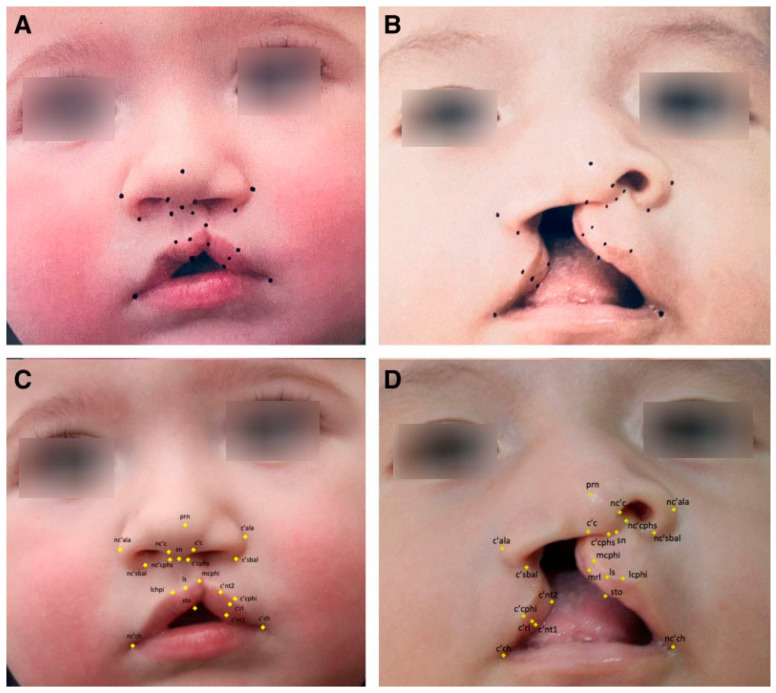
Comparison of hand-marked versus AI-generated unilateral cleft lip markings. (**A**) Right unilateral cleft hand marked by a fellowship-trained cleft surgeon. (**B**) Incomplete unilateral cleft lip hand marked by the same cleft surgeon. (**C**,**D**) AI-generated cleft markings for each of the corresponding hand-marked images. Anthropometric landmarks for unilateral nasolabial repair are indicated. (Adopted from Sayadi et. al., Harnessing the Power of Artificial Intelligence to Teach Cleft Lip Surgery, PRSGO) [21].

Recent advances have allowed for the automation of surgical markings through machine learning, with the goal of reducing variability and enhancing precision in operative design. In our work, we leveraged high-resolution convolutional neural network architectures to train algorithms capable of identifying 21 key anthropometric landmarks on unilateral cleft lip photographs. Training relied on surgeon-annotated datasets, in which each landmark was manually defined by fellowship-trained cleft surgeons. Despite working with relatively modest image volumes compared to larger facial recognition datasets, we achieved normalized mean errors well within accepted AI performance benchmarks and, importantly, consistent with human inter-rater variability [21]. These findings confirm that deep learning can reliably replicate expert surgical annotation in the 2NMD domain (Figure 5).

The next challenge lies in bridging 2D algorithm outputs with the inherently 3D surgical environment. Current algorithms operate on photographs, but operative markings are executed on curved, deformable tissues. By integrating machine-learning outputs with structured light scanning (SLS), 2D landmarks can be translated into accurate 3D coordinates that respect the unique curvature of each patient’s anatomy. This combined workflow would enable AI-generated markings to be projected directly onto the patient using PAR. Importantly, projection does not preclude human oversight: surgeons retain the ability to validate, edit, or override the automated design in real time, effectively creating a collaborative interface between human judgment and algorithmic precision.

Looking forward, the maturation of this paradigm requires large-scale acquisition of 3D surgical datasets. Whereas current algorithms learn from annotated 2D photographs, future models must be trained on diverse repositories of 3D facial scans with surgeon-defined landmarks and operative designs. Such datasets would allow AI to learn not only static anthropometry but also the dynamic changes that occur intraoperatively as tissues are retracted, rotated, or closed. The eventual aim is a surgical co-pilot platform: a closed-loop system where SLS captures patient-specific anatomy, AI algorithms propose operative designs with millimetric fidelity, and PAR projects these guides directly onto the surgical field. In this model, the surgeon is supported by an intelligent, anatomy-aware interface that enhances precision, accelerates training, and enables remote experts to contribute to operative planning and execution anywhere in the world.

## 4. Identified Needs and Global Health Applications

Surgical training remains rooted in the apprenticeship model. This requires prolonged, in-person supervision and iterative skill transfer over years of guided practice. For surgeons seeking to acquire new expertise beyond residency or fellowship, access to education often necessitates costly international travel, extended time away from clinical responsibilities, and immersion in specialized centers of excellence [22,23]. These barriers are burdensome in high-resource countries and prohibitive in low- and middle-income countries (LMICs). In many such regions, there may be few or no structured training programs in subspecialty surgery, and opportunities for mentorship by experienced international faculty are largely dependent on short-term outreach missions. As a consequence, vast populations remain underserved, with millions of patients unable to access even foundational reconstructive procedures that could restore function, appearance, and quality of life.

Cleft lip and palate starkly illustrate this disparity. With an incidence of approximately 1 in 700 live births worldwide, clefts impose profound medical, functional, and psychosocial burdens [24]. Children with untreated clefts often suffer from impaired feeding, recurrent otitis media, speech delay, and lifelong social stigma. In endemic regions where surgical capacity is limited, the backlog of untreated cases continues to grow despite the efforts of well-established outreach organizations. While humanitarian missions provide temporary relief, they often fall short of delivering long-term sustainability. Lasting solutions require empowering local surgeons to perform high-quality cleft repairs independently, thereby embedding expertise within the local healthcare system [25].

Projected augmented reality (PAR) offers promising potential for achieving sustainable skill transfer. By projecting surgical guidance directly onto the patient in the operating room, PAR can provide field-anchored, visually intuitive guidance that is visible to the entire surgical team. Unlike video conferencing or tablet-based AR, which require learners to mentally transpose information from a secondary display onto the operative field, PAR can be seamless. Markings, incision patterns, or anatomical guides would appear exactly where they are needed, on the patient’s own anatomy. When coupled with artificial intelligence algorithms capable of autonomously generating key anthropometric landmarks and incision designs, the system becomes even more powerful. Expert input can be scaled across multiple simultaneous cases, with AI providing a baseline projection that experts can adjust or validate remotely.

This paradigm has the potential to bridge the gap between global surgical need and limited local capacity. By reducing dependence on prolonged co-location with experts, PAR enables remote mentorship that is immediate, context-specific, and sustainable. Compared with headset-based telepresence or VR platforms, projection-based SAR can be deployed with basic light projectors and RGB/RGB-D cameras that are inexpensive, robust, and low-maintenance, thereby reducing hardware cost, bandwidth demands, and training overhead for remote learning.

In the long term, integrating PAR with AI-driven automation may allow expert knowledge to be disseminated rapidly and equitably, equipping in-country surgeons with the tools to deliver reconstructive care at scale. In this way, PAR transforms remote guidance from a descriptive or observational exercise into a hands-on experience, positioning it as a critical enabler in the global effort to close the surgical access gap.

## 5. Limitations and Technical Barriers

Despite its promise, PAR faces several challenges that must be addressed before clinical adoption. Illumination remains a key issue, as the bright surgical lights of the operating room can wash out projected imagery; this can be mitigated with high-lumen projectors or redundant arrays of multiple projectors that collectively increase brightness. Occlusion is another obstacle, as surgeons, assistants, or instruments may inadvertently block the projector’s line of sight. With multi-camera and multi-projector systems that allow for redundancy, we can help ensure that critical overlays remain visible from alternate angles. Registration accuracy is also paramount, since aligning projected images precisely to patient anatomy requires sub-pixel calibration. Encouragingly, recent advances in registration algorithms have markedly improved robustness and made this level of precision feasible with proper calibration workflows. The surface properties of the operative field add further complexity, as wet tissue, blood, and reflective surfaces can distort projected images. Beyond technical hurdles, workflow integration presents a practical barrier: capturing and reconstructing a high-fidelity 3D model of the patient intraoperatively can be time-consuming. Our ongoing work is evaluating novel software pipelines designed to accelerate this process and enable faster digital reconstructions. Finally, the psychological dimension of adoption must not be underestimated. Surgeons will need to become comfortable interacting with novel forms of intraoperative visualization and learn to trust automated guidance. This transition will require rigorous validation studies, iterative user feedback, and dedicated training programs to ensure that PAR is not only technically sound but also embraced as a safe, reliable adjunct to surgical decision-making. Future work should consist of prospective, multi-center trials comparing PAR-assisted cleft lip repair with standard marking—powered for blinded aesthetic and anthropometric symmetry scores, revision rates, operative/anesthesia times, and cost-effectiveness—while quantifying the learning curve with objective proficiency metrics. Embedded sub-studies should evaluate human-factors usability, cybersecurity/PHI safeguards, consent workflows, and implementation feasibility for tele-mentorship in LMIC settings.

## 6. Future Directions

Looking ahead, PAR is poised to evolve into a versatile and intelligent intraoperative platform. Deeper integration with imaging will enable binding of CT and MRI data to the patient surface, allowing critical subsurface structures, such as fracture lines, perforators, or nerves, to be projected directly onto the operative field. Long term, the incorporation of artificial intelligence marks the emergence of PAR as a true surgical “co-pilot,” with algorithms capable of autonomously identifying landmarks, proposing incision designs, and refining intraoperative decisions. With the adoption of federated learning, such systems could continuously improve by learning from global datasets while preserving patient privacy. PAR can expand beyond cleft surgery to a wide spectrum of procedures that rely heavily on surface-based planning and precision, including skin cancer reconstruction, oncologic resections, and neurosurgical procedures, ultimately establishing itself as a universal intraoperative guidance platform across multiple surgical disciplines.

Projected augmented reality represents a paradigm shift in surgical visualization and education. By projecting guidance directly onto the operative field, PAR overcomes many of the limitations of HMD-AR and integrates seamlessly into surgical workflow. Coupled with structured light scanning and artificial intelligence, PAR enables real-time, anatomically precise, and universally visible guidance that can enhance precision in high-resource settings while democratizing expertise globally. PAR anchors guidance directly on the patient, making cues team-visible and loupe/headlight-compatible while removing the cognitive step of translating a headset image to the operative site. These attributes improve mentor–learner concordance at the point of action and lower hardware/infrastructure burden, making the transfer of expertise more scalable—particularly in resource-constrained settings.

As the technology matures, PAR has the potential to transform the operating room into an interactive, intelligent environment; one where expert knowledge, advanced imaging, and machine learning converge at the surgical field. In doing so, PAR can be a tool in expanding access and improving outcomes in surgery globally.

## Supplementary Materials

The following supporting information can be downloaded at: https://www.mdpi.com/article/10.3390/jcm14228246/s1. Video S1: Realignment of Cleft Markings on 3D model.

## Abbreviations

The following abbreviations are used in this manuscript:

AR	Augmented Reality
HMD-AR	Head-mounted Display Augmented Reality
PAR	Projected Augmented Reality
SLS	Structured Light Scanning
